# Single-phase multiferroics: new materials, phenomena, and physics

**DOI:** 10.1093/nsr/nwz091

**Published:** 2019-07-10

**Authors:** Chengliang Lu, Menghao Wu, Lin Lin, Jun-Ming Liu

**Affiliations:** 1 School of Physics & Wuhan National High Magnetic Field Center, Huazhong University of Science and Technology, Wuhan 430074, China; 2 Laboratory of Solid State Microstructures, Nanjing University, Nanjing 210093, China; 3 Institute for Advanced Materials, Hubei Normal University, Huangshi 435002, China

**Keywords:** multiferroics, magnetoelectric coupling, non-reciprocity, topological domain structure, 2D multiferroics

## Abstract

Multiferroics, where multiple ferroic orders coexist and are intimately coupled, promise novel applications in conceptually new devices on one hand, and on the other hand provide fascinating physics that is distinctly different from the physics of high-*T*_C_ superconductors and colossal magnetoresistance manganites. In this mini-review, we highlight the recent progress of single-phase multiferroics in the exploration of new materials, efficient roadmaps for functionality enhancement, new phenomena beyond magnetoelectric coupling, and underlying novel physics. In the meantime, a slightly more detailed description is given of several multiferroics with ferrimagnetic orders and double-layered perovskite structure and also of recently emerging 2D multiferroics. Some emergent phenomena such as topological vortex domain structure, non-reciprocal response, and hybrid mechanisms for multiferroicity engineering and magnetoelectric coupling in various types of multiferroics will be briefly reviewed.

## INTRODUCTION

**Figure 1.**
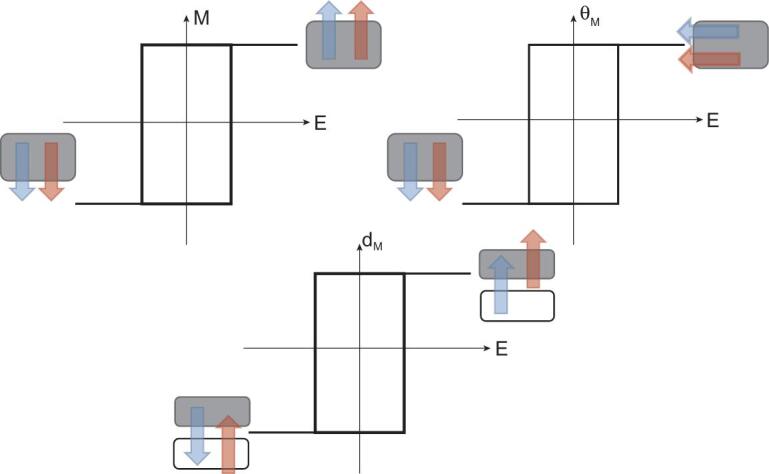
Three types of ME coupling: reversal of net magnetization M upon ferroelectric switching; change of magnetic easy axis upon ferroelectric switching; change of magnetic distribution upon ferroelectric switching in 2D bilayer systems. Red and blue arrows denote the polarization and magnetization directions, respectively. θ_M_ and d_M_ respectively denote the angle of the magnetic easy axis and the center coordinate of magnetic dipoles.

The terminology ‘multiferroics’ refers to those materials where more than one ferroic order, i.e. (anti)ferromagnetism, ferroelectricity, ferroelasticity, and ferrotoroidicity, coexist in one phase [1]. A combination of these multiple ferroic orders may allow intimate coupling among them, paving the way to realizing cross-control of various ordered parameters. The most striking of such controls is switching magnetization and/or ferroelectric polarization by electric and magnetic fields, as illustrated in Fig. 1. Nowadays, the term ‘multiferroics’ is often used specifically for those materials hosting ferroelectric and magnetic behaviors.

Multiferroics have been attracting enormous attention since the publication of the two seminal papers on TbMnO_3_ and BiFeO_3_ in 2003 [2,3]. So far, a large array of materials have been explored to show multiferroicity and magnetoelectric (ME) coupling, and these materials can be roughly classified into categories according to the microscopic mechanisms of ferroelectricity, noting that more than one mechanism may coexist in many cases. As proposed by Khomskii, there are two types of multiferroics: type-I and type-II [4]. Type-II multiferroics, where ferroelectricity is generated in specific magnetic ordering, are sometimes called magnetism-driven multiferroics. All other single-phase multiferroics that do not have a magnetic origin of ferroelectricity can be categorized as type-I multiferroics. To more precisely capture the intrinsic physics, the type-I or type-II families can be further sub-classified according to the detailed microscopic mechanisms for ferroelectricity. This is rather meaningful, especially for type-I multiferroics. The double-layered magnetic perovskites exhibiting hybrid improper ferroelectricity represent an additionally developed sub-class of type-I multiferroics, which have shown highly designable multiferroicity in recent years [5,6].

In addition, while multiferroicity and ME coupling are most frequently discussed as bulk effects, 2D multiferroics and domain wall multiferroicity, which are so far less addressed but definitely appealing, have been revealed recently [7]. Even more, while realization of cross-controls of multiple ferroic orders, which offers routes to entirely new device architectures and may bring a revolution in information processing and storage, has been the main stream of multiferroic research, a number of fascinating phenomena not in the main stream but sufficiently compelling, such as non-reciprocity [8,9], topological orders [10], and thermal Hall effects [11], have been discovered in multiferroic materials. These events allow distinctive functionalities beyond the ME coupling and moisturize multiferroic research.

Concomitant with progress in this highly interdisciplinary field, quite a few review articles on various aspects of multiferroics and underlying physics are available [12–16]. The continuous enthusiastic endeavors on multiferroics have gradually moved this promising discipline into a new era. Especially in the past five years, distinctly different and appreciated multiferroic mechanisms beyond the well-known ones and a series of new phenomena have been observed in several alternative systems, largely extending the essence and scope of multiferroicity. In the present mini-review, we give an overview of the recent advances in three aspects: single-phase materials, emerging phenomena, and updated physical scenarios. We certainly do not make any attempt to cover everything in this short article, and the topics chosen are more or less biased by the authors’ own research interests. The review is organized according to the classification of multiferroics, and only those advances in recent several years in this framework will be discussed.

## TYPE-I MULTIFERROICS

First of all, BiFeO_3_ is an unavoidable material if one discusses type-I multiferroics where ferroelectricity and magnetism are believed to originate from two different cations; i.e. the 6*s* lone pair electrons of Bi give rise to ferroelectricity and the 3*d*-electrons of Fe lead to canted G-type antiferromagnetic (AFM) order [3]. This represents a major strategy that was initially utilized to ‘produce’ type-I multiferroics, and a variety of Bi-based and Pd-based type-I systems have been synthesized and characterized [17]. An extension of this strategy in concomitance with research progress is exemplified by EuTiO_3_ where the 4*f* magnetic moment of Eu and *d^0^*-ferroelectricity of Ti coexist, and are efficiently coupled under some specific conditions [18].

Hexagonal manganites RMnO_3_ (h-RMnO_3_ where R is a rare-earth element or Y, In) serve as another class of materials to obtain type-I multiferroicity, with route in the line of geometric frustration. The buckling of layered MnO_5_ polyhedra that form the in-plane triangle geometry causes the collective movements of Y^3+^ along the *c*-axis with a two-up–one-down profile, leading to the *c*-axis net electric polarization [19]. Since geometrical frustration is essential in generating ferroelectricity, h-RMnO_3_ is also called a geometric multiferroic. A signature of these geometric multiferroics is that both ferroelectricity and magnetism are closely linked to structural distortion, which may promise evident ME coupling through the spin–lattice mechanism. Direct evidence for this is the coupled ferroelectric and magnetic domains in h-YMnO_3_, as observed using the second harmonic generation [20].

**Figure 2.**
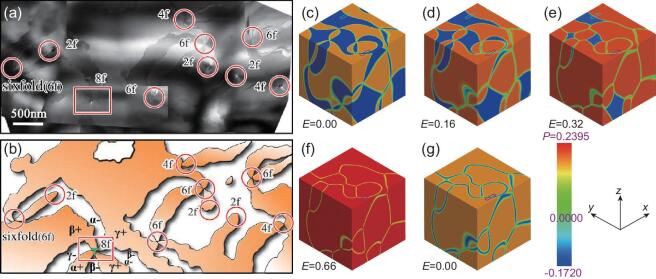
Six-fold and non-six-fold vortex domains in hexagonal YMnO_3_ single crystals: (a) Identified with mesoscale dark-field transmission microscopy, and (b) the corresponding schematic diagram [28]. (c)–(g) illustrate the simulated 3D topological domains manipulated with electric field. The magnitude of ferroelectric polarization is scaled by the color bar [23].

An emerging phenomenon with h-RMnO_3_ is the identification of a unique structurally and ferroelectrically coupled domain structure where six domains in the [α^+^, γ^−^, β^+^, α^−^, γ^+^, β^−^] sequence meet at the conjunct point, constituting the six-fold vortex or antivortex [21]. Each six-fold degenerate vortex pairs with one antivortex and these pairs occupy the whole space, resulting in a topologically non-trivial vortex–antivortex domain structure. The ME coupling mediated by lattice instability at domains and walls can be directly captured using the ME force microscopy technique [22]. In fact, the topological domain structure can further be manipulated by electric field [23,24], stiffness anisotropy [25], and self-poling resulting from oxygen off-stoichiometry [26], as shown in Fig. 2, and also in thin films [27]. The six-fold topological domain pattern may be protected by the so-called Z_2_ × Z_3_ symmetry in h-RMnO_3_, essentially determined by the trimerized tilting of MnO_5_ bipyramids [10], while two-, four-, and eight-fold vortex configurations due to different topological defects have been observed [28], also shown in Fig. 2. Such topological domain patterns are not limited to h-RMnO_3_ but are evidenced in other materials such as BiFeO_3_ [29]. More information can be found in an excellent topic review by Huang and Cheong [10]. In addition, it should be mentioned that the hexagonal structure can be transformed to an orthorhombic one via chemical and strain engineering, and then totally different multiferroicity and ME coupling may be obtained, while orthorhombic RMnO_3_ exhibits type-II multiferroic behaviors, to be illustrated in the section entitled ‘Type-II multiferroics’.

**Figure 3.**
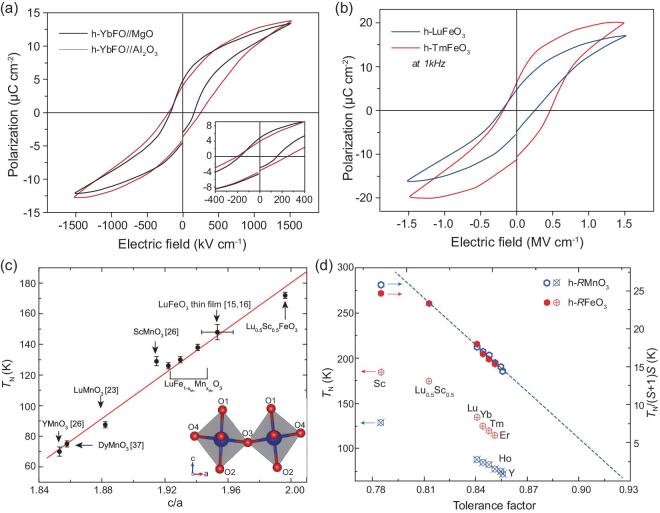
Room-temperature ferroelectric hysteresis loops of (a) hexagonal YbFeO_3_ films grown on different substrates, and (b) hexagonal LuFeO_3_ and TmFeO_3_ films grown on Al_2_O_3_ (0001) substrates [33,34]. Antiferromagnetic transition temperature *T*_N_ as a function of (c) lattice parameter *c*/*a*, and (d) tolerance factor for hexagonal manganites and ferrites [37,38]. In (d), *T*_N_/(*S*+1)*S* as a function of tolerance factor is also plotted, in which *S* means the spin on Fe.

The scenario of geometrically driven multiferroicity is also applicable to other iso-structural compounds, e.g. RFeO_3_ [30–32]. Unlike h-RMnO_3_, hexagonal RFeO_3_ (h-RFeO_3_) is not as stable as orthorhombic RFeO_3_, but the energy difference between them is small, suggesting the possibility of hexagonal ↔ orthorhombic transformation triggered by small external stimuli. For example, h-RFeO_3_ structure can be easily obtained in thin films via the substrate matching route. Indeed, elevated temperature ferroelectricity in (Lu, Yb, Tm)FeO_3_ epitaxial films has been demonstrated and the measured remnant polarization *P*_r_ ∼ 5 μC/cm^2^ [33,34], as shown in Fig. 3. In the meanwhile, switchable ferroelectric photovoltaic effects were revealed in h-RFeO_3_, in analogy to multiferroic BiFeO_3_. Chemical doping is another useful route to achieve a hexagonal phase. Surprisingly, the hexagonal phase can be stabilized by chemical doping at the R-site with a small R-ionic size. One case is (Lu, Sc)FeO_3_ [35], noting that h-LuFeO_3_ is nevertheless chemically unstable. The first-principles calculations on the (Lu, Sc)FeO_3_ solid solution suggested that the hexagonal phase is lower in energy than the orthorhombic phase. This finding is expected to promote efforts to synthesize high-quality h-LuFeO_3_ bulk crystals, once a great challenge. The similar geometric ferroelectricity in h-RFeO_3_ and h-RMnO_3_ hints at the existence of topological domain structure in h-RFeO_3_ [31,36].

In comparison with h-RMnO_3_, h-RFeO_3_ commonly shows much higher antiferromagnetic Néel temperature *T*_N_, appreciated for multiferroic applications. Although the underlying physics remains elusive, the strong Fe–Fe exchange and lattice distortion should be the pivotal ingredients [37], as also found by Sinha *et al_._* that *T*_N_ has a nearly linear dependence on the tolerance factor, suggesting a connection with the polar distortion [38], as shown in Fig. 3. These results offer an efficient strategy to modulate the multiferroic properties via structural engineering.

The final class of type-I multiferroics, which was discovered long ago but recently addressed once more, is fluorides, BaMF_4_ (M = Mn, Ni, Co, Fe, Cu) [39–41]. The ferroelectricity is believed to originate from the collective rotation of MF_6_ octahedra, and transition metals provide magnetism. The ferroelectric Curie temperature in some fluorides can be high, even exceeding 1000 K and the melting point. However, the AFM ordering usually emerges at a much lower temperature, typically *T*_N_ < 50 K. It should be mentioned that multiferroic fluorides have been less addressed as compared with other type-I multiferroics, perhaps due to the difficulties in synthesis of high-quality materials and electric characterization. In addition, strong room-temperature blue–violet photoluminescence in BaMnF_4_ was found very recently [42], a surprising functionality in multiferroic fluorides.

## TYPE-II MULTIFERROICS

For type-II multiferroic materials, electric polarization is believed to be generated by a specific magnetic order, allowing a remarkable ME response. It was initially discovered in orthorhombic RMnO_3_ [2] and RMn_2_O_5_ [43], and subsequently dozens of materials have been found to show type-II multiferroicity. Three microscopic mechanisms, i.e. the inverse Dzyaloshinskii–Moriya (DM) interaction, exchange striction, and spin-dependent *p*–*d* hybridization, have been proposed [12,14]. In particular, for materials hosting spiral spin order (SSO), the electric polarization can be sharply and completely flipped from one direction to another, resulting in remarkable ME response, although the inverse DM interaction-induced polarization is generally small (<0.01 μC/cm^2^) [2]. In contrast, the exchange-striction-induced electric polarization, most likely observed in materials with collinear spin order, can be much larger (>0.1 μC/cm^2^), and even comparable with conventional ferroelectrics [44,45]. Recently, a giant polarization of *P* ∼ 2 μC/cm^2^ in RMnO_3_ under pressure, due to exchange striction, was demonstrated [46–48]. It should be mentioned that the ME coupling in multiferroics dominated by the exchange striction mechanism is usually not as striking as that in multiferroics of spiral spin order, and polarization switching usually requires a relatively high magnetic field. It is noticed that the spin-dependent *p*–*d* hybridization mechanism is found only in several materials although a unique ME coupling mode that the electric polarization can be successively rotated by magnetic field has been observed [49].

The past five years have seen the appearance of many more type-II multiferroics; it would be impossible to mention all of them, but several examples are described below.

1. Aeschynite-type polar magnets RFeWO_6_ that possess multiferroicity with spontaneous polarization *P* ∼ 3 μC/m^2^ below ∼20 K, arising from an unusual commensurate and non-collinear magnetic configuration of Fe^3+^ [50]. Further theoretical calculations revealed a polarization as large as ∼ 7.5 μC/cm^2^, likely accessible in single crystals.
2. Tungstate LiFe(WO_4_)_2_ with a wolframite structure. A polarization of ∼10 μC/m^2^ due to non-collinear magnetism of Fe^3+^ in polycrystalline samples below ∼20 K was reported [51]. The spiral spin order-driven multiferroicity looks highly sensitive to structural variations, noting that NaFe(WO_4_)_2_ is non-multiferroic in spite of its similarity to LiFe(WO_4_)_2_ in composition and stoichiometry but its difference from LiFe(WO_4_)_2_ in crystallographic structure. The high sensitivity to lattice structure suggests spin–lattice coupling as the possible mechanism for the distinctive ME controls.
3. Vanadate M_2_V_2_O_7_, another family that was found to show type-II multiferroicity. In (Ni, Co)_2_V_2_O_7_, two multiferroic phases were respectively identified at low and high magnetic field regions, while a non-ferroelectric phase in between them with a half-magnetization plateau was observed [52]. These observations are similar to the measured results on Ni_3_V_2_O_8_ and MWO_4_, and the existence of multiple multiferroic phases may be utilized for unusual ME memory applications [53,54].
4. A long list of other type-II multiferroics, including α-NaFeO_2_ [55], Mn_2_O_3_ [56], (La, Bi)Mn_3_Cr_4_O_12_ [57,58], Ni_3_TeO_6_ [59,60], (Fe, Mn)_2_Mo_3_O_8_ [61–63], Co_4_Nb_2_O_9_ [64], Mn_2_MnWO_6_ [65], RFe_3_(BO_3_)_4_ [66–68], KCu_3_As_2_O_7_(OD)_3_ [69], NaFeSi_2_O_6_ [70], In_2_NiMnO_6_ [71], and so on. Certainly, this list should not exclude materials other than oxides, and compounds with other anions such as S and Se have been revealed to show type-II multiferroicity too, including CaOFeS [72], MnSb_2_S_4_ [73], BaFe_2_Se_3_ [74], Cu_3_Bi(SeO_3_)_2_O_2_Cl [75], CsCuCl_3_ [76], and CuBr_2_ [77].

Although the physics of most type-II multiferroics can be understood within the framework of these three mechanisms, there are exceptions, at least qualitatively. One exception is LaMn_3_Cr_4_O_12_,

a system with cubic quadruple perovskite structure and collinear spin alignment, and the spin-driven ferroelectricity cannot be ascribed to any of the three mechanisms [58]. A scenario composed of spin–orbit coupling and dual G-type AFM ordering was proposed to interpret the multiferroicity. Apart from this, it was demonstrated that both ferroelectric polarization and ME coupling can be significantly enhanced if La is replaced by the well-known ferroelectric active cation Bi^3+^. BiMn_3_Cr_4_O_12_ provides a rare example with joint multiferroicity in the same phase [57]. Spin-ice pyrochlores such as Ho_2_Ti_2_O_7_ and Dy_2_Ti_2_O_7_, highly noted for their unusual finite zero-point entropy and magnetic monopoles, were recently found to host multiferroicity and ME coupling, which probably has a spin-related mechanism [78–80].

**Figure 4.**
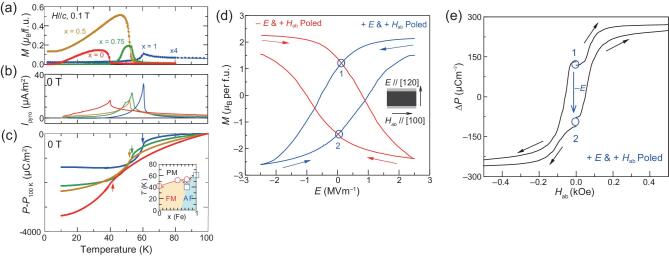
Temperature dependence of (a) magnetization, (b) pyroelectric current, and (c) time-integrated change of polarization from that at *T* = 100 K in multiferroic (Mn_1-x_Fe_x_)_2_Mo_3_O_8_ [62]. In the inset of (c), a summarized phase diagram is presented, in which the open circles and squares are estimated transition temperatures. (d) Electric reversal of magnetization, and (e) *P*-variation as a function of magnetic field at *T* = 10 K in hexaferrite Ba_0.4_Sr_1.6_Mg_2_Fe_12_O_22_ [81].

Despite these advances in the past two decades, indomitable effort in searching for more compounds with desirable performance is still underway. Room-temperature multiferroicity, sufficient ME response, and ferromagnetism, as well as electro-control of magnetization, remain the major concerns. One may find that antiferromagnetism and spin-driven ferroelectricity look like the two sides of a coin for type-II multiferroics. Regarding this, building up a ferrimagnetic lattice seems to be a promising route to have evident net magnetization, accessed in the M_2_Mo_3_O_8_ family where sizeable macroscopic magnetization ∼0.5 μ_B_/f.u. and ferroelectric polarization as large as ∼0.2500 μC/cm^2^ have been measured [62], as shown in Fig. 4. The electrically driven reversal of a large magnetization of ∼2.0 μ_B_/f.u in Y-type hexaferrite Ba_0.4_Sr_1.6_Mg_2_Fe_12_O_22_ was identified, accompanied with a giant ME coefficient ∼33 000 psm^−1^ [81], as shown in Fig. 4 too. Theoretically, near-room-temperature tunable ferromagnetism and electric polarization as large as ∼10 μC/cm^2^ in R_2_NiMnO_6_ superlattices have been predicted, definitely deserving experimental exploration [82].

**Figure 5.**
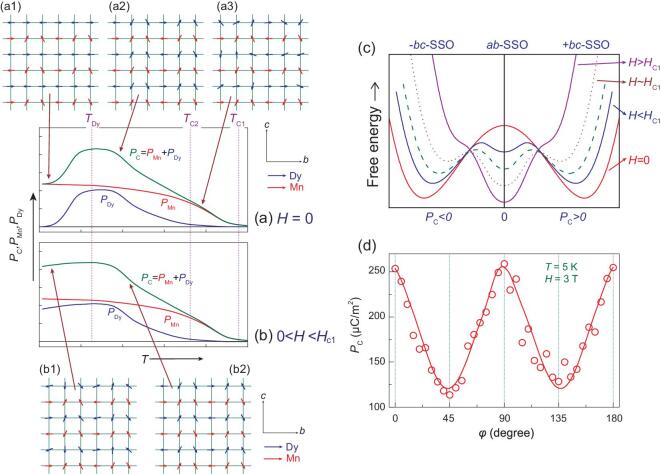
Schematics of the dual nature of multiferroicity in orthorhombic DyMnO_3_. (a) At *H* = 0, *P*_Mn_ ∼ *S*_Mn_ × *S*_Mn_ arises at *T*_C1_, and then *P*_Dy_ ∼ *S*_Dy_•*S*_Mn_ emerges at *T*_C2_ due to the Dy–Mn exchange striction. Below *T*_Dy_, *P*_Dy_ is diminished with the onset of an independent Dy spin order. (b) With *H* < *H*_c1_ (before the flip transition of the spiral plane), *P*_Dy_ can be recovered due to the re-emergence of Dy–Mn coherence. (c) Sketch of an energy diagram of *P*-switching and the corresponding spin-spiral plane in DyMnO_3_, which can be tuned by the application of *H*. (d) Continuous magnetoelectric control in DyMnO_3_ films with twin-like domains, which can be qualitatively understood using the energy diagram shown in (c) [85].

As stated earlier, the inverse DM interaction can generate a remarkable ME response, while the exchange striction can provide large electric polarization. It has been a long-standing issue to combine the two mechanisms concurrently in the same system. This issue was once investigated taking orthorhombic DyMnO_3_ as an example. It was proposed that the electric polarization consists of two components, including component *P*_Mn-Mn_ due to the inverse DM interaction with Mn^3+^ spiral spin order and component *P*_Dy-Mn_ due to the Dy^3+^–Mn^3+^ exchange striction [83–88], as illustrated in Fig. 5. Component *P*_Mn-Mn_ can be flipped by magnetic field *H* from one direction (i.e. the *c*-axis) to another (i.e. the *a*-axis). Importantly, component *P*_Dy-Mn_ rigidly tracks component *P*_Mn-Mn_, and thus remarkably enhances the total electric polarization and ME effect. We may call this behavior the hybrid mechanism. In addition, if a twin-like structural domain structure is involved in DyMnO_3_ thin films, a distinctive continuous ME control has been observed [85], as shown in Fig. 5 too. Such a hybrid mechanism is not limited to DyMnO_3_ but is broadly available in the orthorhombic RMnO_3_ family, representing another efficient roadmap to enhance the multiferroicity and ME coupling. Usually, this hybrid mechanism requires more than one magnetic cation, such as Dy and Mn in DyMnO_3_, compatible with the scenario of magnetism enhancement in a ferrimagnetic lattice.

**Figure 6.**
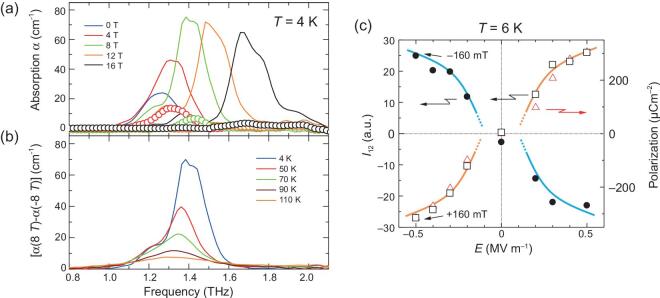
(a) and (b) present the remarkable difference of absorption coefficient for positive (solid lines) and negative (open circles) magnetic fields in (Fe_0.6_Zn_0.4_)_2_Mo_3_O_8_ [91]. (c) Non-reciprocal microwave response in a multiferroic helimagnet Ba_2_Mg_2_Fe_12_O_22_ [93]. The integrated intensities of non-reciprocity *I*_12_ can be reversed by an external electric field at *T* = 6 K, consistent with the *P*-switching behavior (open triangles).

Besides good materials for multiferroicity, emergent phenomena discovered in type-II multiferroics are also receiving attention. For example, non-reciprocity and the thermal Hall effect observed in some multiferroics are indeed compelling [8,9]. The non-reciprocal effect means that the motion of an object such as an electron, magnon, phonon, and light in one direction is different from that in the opposite direction, like the diode effect. The crucial physical ingredient for a non-reciprocal effect is broken inversion symmetry. In the case of multiferroics, both time reversal and space inversion symmetries are broken, and thus non-reciprocal directional dichroism/birefringence can in principle be widely observed. For instance, one-way transparency of light was revealed in multiferroic CuBO_2_ [89], and magnetic switching of light transmission was identified in (Cu, Ni)B_2_O_4_ [90]. In FeZnMn_3_O_8_, a giant terahertz optical diode effect was reported even in the paramagnetic phase [91], as shown in Fig. 6. In fact, a room-temperature optical diode effect due to the spin-current-driven dynamic ME coupling was demonstrated in BiFeO_3_ [92]. More interestingly, the non-reciprocal microwave response in multiferroic helimagnet Ba_2_Mg_2_Fe_12_O_22_ can be reversed by an electric field [93], as shown in Fig. 6 too. While these investigations have illustrated the tantalizing aspects of non-reciprocity in multiferroics, more investigations are desired to exploit the full merit of this effect, such as efficient controls of the significantly large non-reciprocal response by means of an electric/magnetic field above room temperature, and readers may refer to two recent topic review papers [8,9].

Type-II multiferroics (especially those dominated with the exchange striction mechanism) usually show strong spin–lattice coupling, which guarantees the cross-control of phonons (spin excitations) by a magnetic field (electric field). The thermal Hall effect is one typical example, in which the heat flow dominated by phonons can be deflected by a perpendicular magnetic field. Unlike charged particles or quasi-particles, the phonon is neutral. In (Zn*_x_*Fe_1-_*_x_*)_2_Mo_3_O_8_, a giant thermal Hall effect *k_xy_*∼ 30 × 10^−3^ W K^−1^m^−1^ was observed, making an effective probe for spin–lattice coupling and magnetic control of thermal currents possible [11]. Certainly, one may ask how a thermal Hall effect can be effectively modulated electrically, and whether the flow of phonons possesses any non-reciprocal response.

## HYBRID IMPROPER MULTIFERROICITY IN DOUBLE-LAYERED PEROVSKITES

It is still not very clear what the third road is, in addition to type-I and type-II multiferroics, considering the fact that none of these materials can meet all requirements for multiferroicity. Nevertheless, improper ferroelectrics with magnetic species seem to be very interesting recently. For perovskites ABO_3_, rotation and tilting of BO_6_ octahedra can be ubiquitously observed if the tolerance factor is small. This is a kind of antiferrodistortion (AFD) mode, leading to a local charge dipole associated with the bent B–O–B bonds.

**Figure 7.**
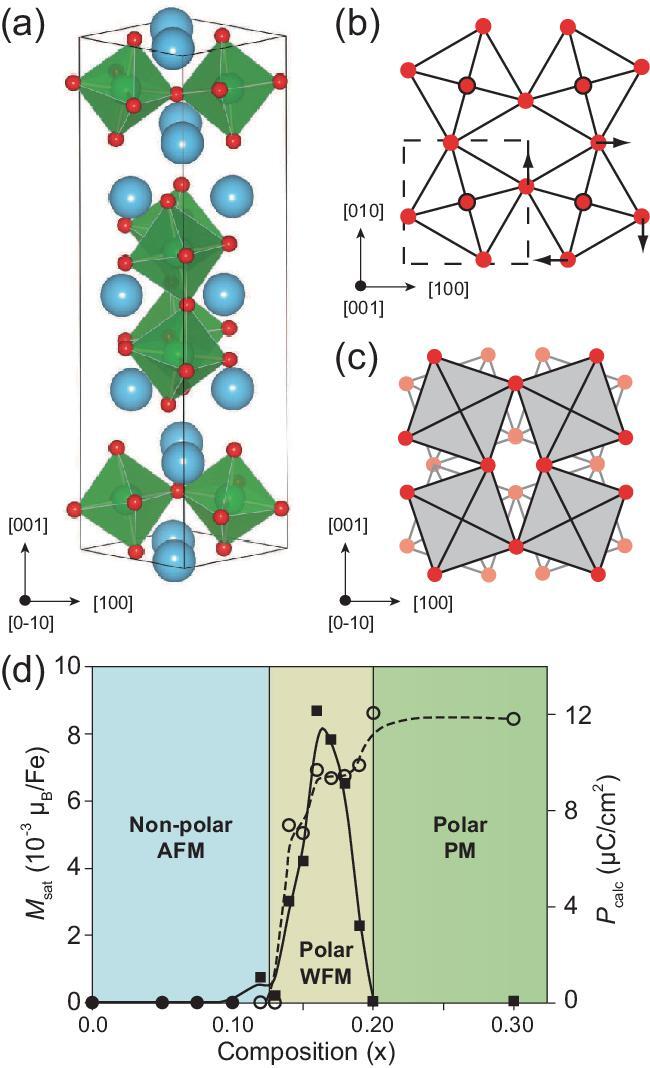
(a) Crystal structure of double-layered perovskite A_2_B_3_O_7_ showing octahedral tilting and rotation. Two different antiferrodistortive modes: (b) the *X*_2_^+^ rotation mode, and (c) the *X*_3_^−^ tilt mode [5]. (d) Phase diagram of (1-*x*)(Ca_0.6_Sr_0.4_)_1.15_Tb_1.85_Fe_2_O_7_ –(*x*)Ca_3_Ti_2_O_7_ at *T* = 300 K [97]. Solid squares: measured saturated magnetization. Open circles: calculated polarization.

Unfortunately, such local charge dipole in these simple perovskites would be compensated by the neighboring BO_6_ layers, resulting in zero net electric polarization, as often observed. An extension of this scenario towards double-layered perovskites A_3_B_2_O_7_ would make the story different. Here, a combination of two AFD modes would generate an improper electric polarization [5], already demonstrated in a large array of compounds such as Ca_3_Ti_2_O_7_ [6,94,95], Ca_3_Mn_2_O_7_ [96], and the (1-*x*)(Ca_0.6_Sr_0.4_)_1.15_Tb_1.85_Fe_2_O_7_–(*x*)Ca_3_Ti_2_O_7_ series [97] etc., as shown in Fig. 7. In this sense, improper magnetic ferroelectric compounds, tentatively called hybrid improper multiferroics, offer opportunities with which one may find some good multiferroics. Surely, one may argue that this two-mode combination is similar to the physics of type-I multiferroics where ferroelectricity and magnetism have separate origins. The striking feature of these hybrid improper multiferroics is that ferroelectricity and magnetism arise from the same cation, allowing prominent ME control via spin–lattice coupling.

Hybrid improper multiferroics show at least three advantages, evidenced in several double-layered perovskites. First, both ferroelectric and magnetic transition may occur above room temperature, pivotal for potential applications. Weak ferromagnetism and polar phase were revealed above *T* ∼ 300 K in (1-*x*)(Ca_0.6_Sr_0.4_)_1.15_Tb_1.85_Fe_2_O_7_–(*x*)Ca_3_Ti_2_O_7_ with 0.1 < *x* < 0.2 [97]. Second, double-layered perovskites A_3_B_2_O_7_ are highly designable by simple chemical consideration. Both the A-site and B-site can be occupied by either different cations or cation groups, providing a broad platform to engineer the physical properties on purpose. Third, multiferroicity in A_3_B_2_O_7_ is simply due to structural distortion; i.e. a combination of non-polar rotation of BO_6_ octahedra induces net electric polarization. Along this line, a natural proposal to tailor the multiferroicity of A_3_B_2_O_7_ is thin film epitaxy. An ultra-low coercive field for ferroelectric switching was identified in Ca_3_Ti_2_O_7_ epitaxial films, although the bulk counterpart shows a huge coercivity ∼200 kV/cm [98].

A more interesting concept along this way is an integration of the type-II mechanism into hybrid improper multiferroics. Because of the clear advantages of the A_3_B_2_O_7_ family for highly tunable chemical compositions, comprehensive chemistry engineering makes it possible to design special magnetic configurations that could generate type-II multiferroicity. In this sense, to simultaneously obtain good ferroelectricity, giant ME coupling, and high ferroelectric/magnetic transition temperatures in a single material seems to be beyond a dream.

## OTHER SINGLE-PHASE MULTIFERROICS

While type-I, type-II, and hybrid improper multiferroics represent the three main streams, there are also many brooks that may not be significant but are interesting. We highlight several cases without losing the generality.

First, a common perception assumes that a ferroelectrically active cation has to follow the *d^0^*-rule. As an exception, non-*d^0^* Mn-driven ferroelectric displacement was initially proposed in antiferromagnetic BaMnO_3_ [99], and subsequently demonstrated in Sr_1-_*_x_*Ba*_x_*MnO_3_ [100] and SrMnO_3_ [101]. The non-*d^0^* ferroelectricity originates a spontaneous off-center displacement of Mn, arising from a second-order Jahn–Teller distortion. In Sr_0.5_Ba_0.5_MnO_3_, room-temperature ferroelectricity was evidenced, and a large spontaneous polarization ∼4.5 μC/cm^2^ was obtained at *T* = 2 K (which could be triple as the sample is single-domained) [100]. In strained SrMnO_3_ thin films, a well-defined ferroelectric hysteresis loop with a gigantic remnant polarization of ∼55 μC/cm^2^ at *T* = 10 K was reported very recently [101].

Second, theoretical works have suggested that the non-*d^0^* Mn-driven multiferroicity is highly tunable. A rich multiferroic phase diagram was proposed in strained SrMnO_3_ thin films, and coexistence of ferromagnetism and ferroelectricity was found as the strain was above ∼4% [102]. By replacing Sr with Ba in strained thin films, a first-order magnetic transition, i.e. from the AFM state with smaller polarization to the ferromagnetic state with larger polarization, was predicted [103]. Subsequently, the *ab initio* calculations predicted a strain-induced morphotropic phase boundary (MPB) in Sr_0.5_Ba_0.5_MnO_3_ thin films accommodating giant ME coupling and magneto-striction [104]. The multiferroicity due to the non-*d^0^* cation off-center displacement was observed in super-tetragonal BiFeO_3_, and scanning transmission electron microscopy imaged a relative displacement between Fe and Bi ions as large as ∼ 0.33 Å [105]. Such a super-tetragonal phase can be switched to rhombohedral phase, suggesting a striking modulation of physical properties [106]. In addition, perovskite RCrO_3_ (and probably the RCrO_4_ family) represents another group of multiferroics that show ferroelectricity due to the non-*d^0^* cation off-center displacement, and this topic is nevertheless much less addressed [107,108]. These findings also suggest structural instability in non-*d^0^* cation oxides if the cation–O–cation bond is largely elongated, favoring an off-center displacement. This scenario resembles conventional ferroelectrics, thus hinting at two possibilities for non-*d*^0^ multiferroicity: high strained epitaxy and negative chemical pressure with a big A-site cation, as exemplified in Sr_0.5_Ba_0.5_MnO_3_ [100].

Third, there are more materials that have been demonstrated to possess multiferroicity but the underlying mechanism remains elusive. For instance, *ϵ*-Fe_2_O_3_ was theoretically proposed to show room-temperature multiferroicity with coexisting ferrimagnetism and ferroelectricity [109]. Room-temperature ferroelectricity and ferromagnetism were identified in SrFeO_2.5_, probably due to the displacement of ions and rotation of tetrahedra [110]. In fact, even for those multiferroics whose properties are believed to be well understood, some unexpected phenomena are often observed if a more detailed investigation is carried out. One example is GdMn_2_O_5_, a member of the intensively studied RMn_2_O_5_ family, and the multiferroicity can be notably tuned by electric field poling in the paraelectric phase, an amazing fact to be understood [111].

## 2D MULTIFERROICS

Dimensionality is a well-accepted and promising concept that often makes major impact on materials properties and underlying physics. This concept is also significant for multiferroics, and thus 2D magnetism and ferroelectricity have been investigated recently. The existence of 2D ferromagnetism represents a major breakthrough in condensed matter physics, stimulating strong interest in 2D ferroelectricity and multiferroicity. Traditional ferroelectrics/multiferroics like perovskites with large band gap and low carrier mobility are usually discussed in the 3D framework where dimensionality is not a critical issue. For a 2D van der Waals material, the ferroelectric instability thus becomes of interest, considering the fact that the size effect of ferroelectricity was once one of the central issues for the physics of ferroelectrics due to the depolarization field [112–115]. Such depolarization field effect in a true 2D system may no longer be an issue.

For application consideration, atomically thin ferroelectrics, if any, would make ultra-high-density data storage possible. The van der Waals interaction at the interface between 2D materials and 3D semiconductors also allows lattice mismatch for epitaxial growth, while many 2D materials are already known to be semiconductors with high mobility that may become the basic materials for future wafers. Although research on 2D multiferroics is still at the early stage, and most reports are purely theoretical designs [116], the results available so far on the ME coupling in 2D systems are diversified but the scheme of ‘magnetic reading + electrical writing’ may be more easily accessed in 2D multiferroics.

**Figure 8.**
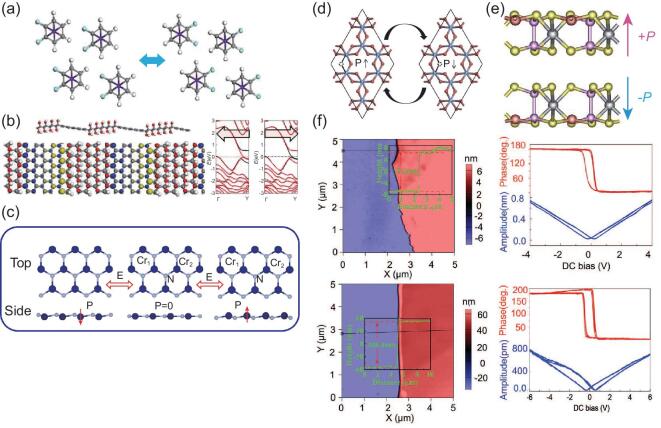
(a) Multiferroicity induced by partial functionalizations of ligand-like halogen on quasi-1D transition-metal-molecular-sandwich nanowires, where the polarization can be reversed upon the rotation of polar nanowires [117]. (b) Multiferroic periodically hydroxylized graphene nanorods, where the polarization of hydroxylized regions can control the spin-splitting of edge states [119]. (c) Vertical ferroelectric in magnetic CrN induced by off-centering atomic displacements of Cr atoms [121]. (d) Ferroelectric switching upon the migration of I vacancy in CrI_3_ [123]. (e) Prediction of ferroelectric switching upon the off-centering displacement of Cu atoms in monolayer CuCrP_2_X_6_ [124], and (f) experimental measurement of the out-of-plane ferroelectricity in few-layer CuCrP_2_S_6_ [125].

It has been predicted that many 2D multiferroics are of type-I characters with independent magnetism and ferroelectricity. The covalent functionalization may induce ferroelectricity, and thus multiferroicity may be obtained by functionalization of nanomagnets. First, magnetic quasi-1D transition metal and molecule sandwiched nanowires, as shown in Fig. 8a, can also become ferroelectric via partial functionalization that can transform the sandwiched benzenes into difluorobenzene, dichlorobenzene, dicyanobenzene, etc. The broken inversion symmetry allows a horizontally switchable polarization [117]. The first 2D multiferroic proposed in 2013 was based on such an approach, as shown in Fig. 8b: half-hydrogenated graphene (graphone) was known to be ferromagnetic, and it can be ferroelectric if the hydrogen atoms are substituted by hydroxyl groups [118]. Here, the in-plane electric polarization stems from the displacement of protons as the hydroxyl groups form into a hydrogen-bonded network, which is completely independent of the magnetism induced by unpaired electrons. Another design of periodically hydroxylized graphene [119] is shown in Fig. 8b, where the graphene nanorods are divided by hydroxylized regions with two magnetic zigzag edges that are antiferromagnetically coupled. The energy degeneracy of the two spin-polarized edge states can be broken by ferroelectric polarization of the hydroxylized regions, and the polarization direction will determine which spin-channel is metallic or insulating. Similarly, CH_2_OCH_3_-functionalized stanene has already been predicted to be ferroelectric [119], and another theoretical work demonstrated the coexistence of ferroelectricity and ferromagnetism in germanene that is half-side passivated by –CH_2_OCH_3_ [120]. The in-plane polarization (∼0.8 × 10^−10^ C/m) can be switched by a rotation of ligands with a low kinetic barrier of ∼0.1 eV, and the unoccupied *p_z_* orbits on the non-passivated Ge sites give rise to ferromagnetism with a magnetic moment of ∼1.0 μ_B_ per atom.

The coexistence of 2D ferromagnetic and ferroelectric states was also predicted in buckled CrN and CrB_2_, where the out-of-plane electric polarization is induced by off-centering atomic displacements of Cr atoms while the magnetism stems from the Cr atoms too, as shown in Fig. 8c [121]. Similarly, a VOCl_2_ monolayer was predicted to possess a large intrinsic in-plane spontaneous polarization of 312 pC/m and stable antiferromagnetism with a Néel temperature as high as ∼177 K [122]. The V off-center displacement that contributes to ferroelectricity can be ascribed to the pseudo Jahn–Teller distortion, while the magnetism stems from the V ion too. Additionally, the coexistence of intrinsic ferromagnetism and switchable out-of-plane polarization induced by I vacancies in CrI_3_ was revealed, where I vacancies may hop between the top and bottom surfaces easily, thereby bringing about a moderate energy barrier for polarization switching [123], as shown in Fig. 8d.

Recently, multiferroicity in the transition metal thiophosphate (TMTP) family has also been predicted. For example, monolayer CuMP_2_X_6_ (M = Cr, V; X = S, Se) is both ferroelectric and ferromagnetic [124]. The out-of-plane electric polarization is induced by the Cu off-center displacement while the magnetism stems from the Cr/V atoms, as shown in Fig. 8e. Experimental observation of the out-of-plane ferroelectricity in few-layer CuCrP_2_S_6_ (approximately 13 nm in thickness) at room temperature was reported later; see Fig. 8f [125]. The 2D ferromagnetism of the few layers was inferred from the magnetic hysteresis of massively stacked nanosheets at 10 K.

**Figure 9.**
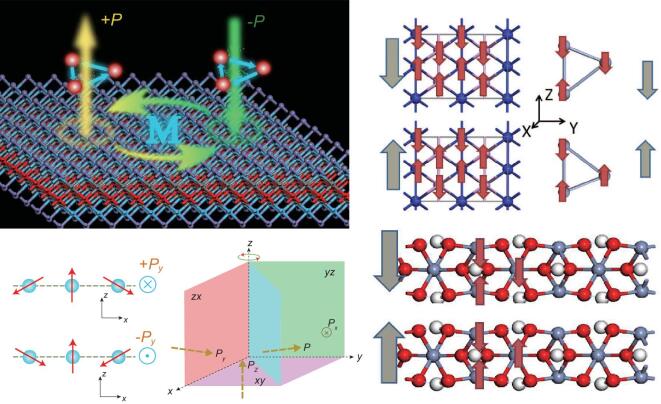
Type-II multiferroicity in MXene Hf_2_VC_2_F_2_ monolayer [126] (left) and in bulk/thin-layer CrOOH [127] (right), where the red arrows denote spin directions and olive arrows denote polarization directions.

To our knowledge at present, MXene Hf_2_VC_2_F_2_ monolayer is one of only two reported 2D type-II multiferroics [126], where the ferroelectricity originates directly from its magnetism, as shown in Fig. 9. The non-collinear 120° Y-type spin ordering breaks the inversion symmetry and generates a polarization perpendicular to the spin helical plane. Remarkably, the estimated multiferroic transition point can be above room temperature, while its polarization is about ∼ 2.9 × 10^−7^ μC/m, much smaller than most 2D ferroelectrics. In another prediction, a combination of type-I and type-II multiferroic modes was revealed in CrOOH, where the switchable polarization was estimated to be 1300 μC/m^2^ [127]. The out-of-plane polarization of 1.2 × 10^−5^ μC/m appears in a thin layer of CrOOH isolated from the (001) surface. This CrOOH layer is also ferrimagnetic with a net magnetic moment as large as 3.0 μ_B_ per unit cell that can be reversed upon the ferroelectric polarization switching.

**Figure 10.**
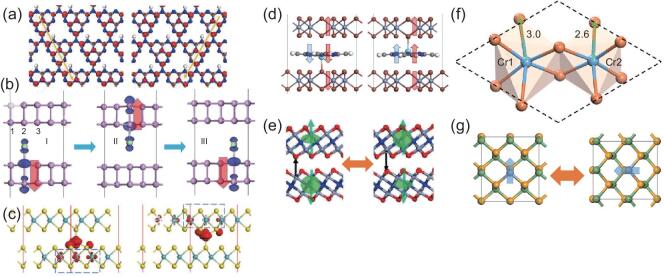
The change of magnetic distribution (in red or blue) upon ferroelectric switching in 2D multiferroics: (a) C_6_N_8_H organic network [128], (b) hydrogen/halogen intercalated 2D van der Waals bilayer [129], and (c) Co-intercalated bilayer MoS_2_ [130]; reversal of net magnetization upon ferroelectric switching in 2D multiferroics: (d) MP intercalated bilayer CrI_3_, [131] and (e) bilayer MXene, VS_2_, and MoN_2_ [132]; the change of magnetic easy axis upon (f) 120 degree ferroelectric switching in charged CrBr_3_ monolayer [133] and (g) 90 degree ferroelectric switching in Cr-doped GeSe monolayer [134].

For many reported 2D multiferroics, ferroelectricity can be used to control the spin distribution despite it not directly stemming from magnetism. A recent theoretical study [128] predicted that the C_6_N_8_H organic network synthesized through hydrogenation of g-C_3_N_4_ is a 2D organic multiferroic with coupled ferromagnetism and ferroelectricity. Its ferroelectricity stems from the proton-transfer within the 2D organic network, which can be used to control the magnetism as the protons make the spin-density distribution anisotropic. As shown in Fig. 10a, the spin-density distribution will be alerted by proton-transfer FE switching.

Another first-principles calculation [129] predicted the evidence of coupled ferroelectricity and ferromagnetism in halogen-intercalated phosphorene bilayer: the ‘mobile’ magnetism can be controlled by ferroelectric switching upon external electric field, exhibiting either an ‘on’ state with spin-selective and highly *p*-doped channels, or an ‘off’ state insulating for both spin and electron transport in the bottom layer attached by all electrodes. The out-of-plane ferroelectric polarization can be maintained against the depolarization field, rendering high-density data storage possible. Moreover, all these functionalities in the halogenated regions can be directly integrated into a 2D phosphorene wafer, similar to the *n/p* channels in a doped silicon wafer. Herein the ferroelectricity is induced by halogen adatom hopping between neighboring bilayers, as shown in Fig. 10b, where the hopping from the upper layer to the lower layer will also ‘transfer’ the magnetic moment from the up state to the down state. The intercalation of halogen or hydrogen adatoms may be extended to other van der Waals bilayers like graphene for the generation of 2D out-of-plane ferroelectric polarization.

Similar ME coupling in metal-intercalated MoS_2_ bilayer has been predicted. Taking Co_0.028_MoS_2_ as an example [130], the ferromagnetic ground state is 55 meV per unit cell lower in energy than that of the antiferromagnetic state, revealing the robust ferromagnetism at ambient conditions. During the switching from state I to state II, a net charge around 0.04e is transferred from the upper layer to the lower layer. The upper layer is mostly spin non-polarized when the electrical polarization points downwards in state I, but becomes spin polarized as the polarization is reversed into state II, and vice versa for the lower layer, as marked by the spin densities in Fig. 10c. As a result, the ferroelectricity and magnetism can be coupled, where the spin distribution can be controlled by the electric field, rendering efficient ‘magnetic reading + electrical writing’.

There have also been reports that the net magnetization of 2D multiferroics can be reversed by ferroelectric switching. When a metal porphyrin (MP) molecule is intercalated between two CrI_3_ layers, the metal ion will be bound to either side [131]. As a result, a double-well potential can be created, which gives rise to a switchable out-of-plane polarization of ∼0.08 eÅ with the barrier lower than 0.35 eV. Since CrI_3_ has been verified to be intralayer ferromagnetic and interlayer antiferromagnetic, the net magnetization of bilayer CrI_3_ should be zero. Upon the intercalation of magnetic MP molecules, however, both TiP/VP will be ferromagnetically coupled to the binding layer. Each TiP and VP molecule possesses a magnetic moment of ∼2.0 μ_B_ and ∼3.0 μ_B_, respectively, and the intercalated bilayer systems keeps the same net magnetization, which can be reversed as the MP binding to the other side upon ferroelectric switching, as displayed in Fig. 10d.

In another work, a series of van der Waals bilayers (BN, MoS_2_, etc.) have been predicted to be 2D ferroelectrics with an interlayer voltage [132], while some magnetic bilayers such as MXenes, VS_2_, MoN_2_, and LaCl/LaBr are multiferroic. For two monolayers that are antiferromagnetically coupled in the ground state, which are inequivalent due to the interlayer charge transfer and voltage difference, their total magnetic moments may be a non-zero value upon an incomplete compensation. The total magnetization can be switched with the reversal of polarization and interlayer voltage, as shown in Fig. 10e, and the predicted switchable magnetizations for bilayer MXene, VS_2_, and MoN_2_ are respectively 0.008, 0.016, and 0.09 μ_B_ per unit cell.

In some 2D multiferroics where the easy axis of magnetism is coupled with the electric polarization direction, the magnetization may rotate by an angle as the ferroelectric polarization switches to another axis. A first-principles study [133] revealed that a charged CrBr_3_ monolayer exhibits the in-plane multiferroicity with coexisting ferromagnetism and ferroelectricity, which is ensured by the combination of orbital and charge ordering as realized by the asymmetric Jahn–Teller distortions of octahedral Cr –Br_6_ units, as displayed in Fig. 10f. The easy axis of magnetization is within the atomic plane and parallel to the in-plane polarization, so the magnetization will be switched by 120 degrees upon a 120 degree ferroelectric switching. In another study [134], 2D triferroics (ferroelectricity + ferromagnetism + ferroelasticity) can even be obtained by doping 3*d* transition metal ions in group-IV monochalcogenide monolayers, which were previously revealed to be ferroelastic multiferroics [135,136]. For some Cr-doped systems including (Cr)SnS, (Cr)GeSe, and (Cr)SnSe, their easy axis of magnetization is aligned along the *x*-axis in-plane, which will switch to the *y*-axis upon either ferroelastic switching or 90 degree FE switching due to the swapping of zigzag and armchair direction, and vice versa if the easy axis is aligned along the *y*-axis, as displayed in Fig. 10g. As a result, upon ferroelastic switching or 90 degree FE switching, their magnetization direction will also switch by 90 degrees, so the ferromagnetism, ferroelectricity, and ferroelasticity are all coupled for efficient data reading and writing.

## FINAL REMARKS

To summarize, we have presented a brief overview on the recent progress of single-phase multiferroics, and it is shown that multiferroicity is still one of the hottest topics in condensed matter physics and materials sciences. Given the rapidly developing nature of this discipline and the authors’ personal bias, it is inevitable that some important papers on this topic have been missed in the present review. In the past five years, tremendous progress has been made in understanding materials chemistry, multiferroicity, and ME coupling. However, the search for new multiferroic materials with good properties, i.e. high temperature, large polarization, strong magnetization, and significant ME coupling, is still underway.

**Figure 11.**
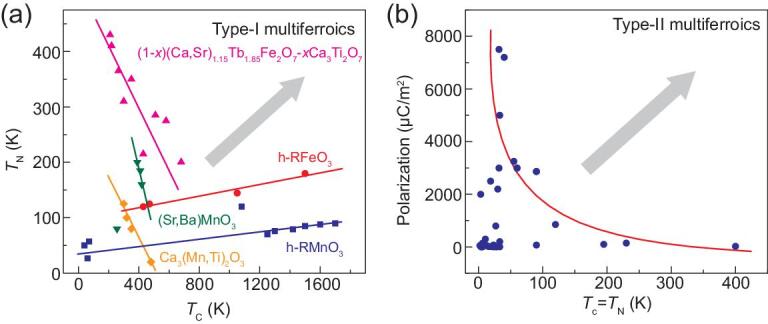
A brief summary of physical properties such as Néel point *T*_N_, ferroelectric transition *T*_C_, and polarization for (a) type-I and (b) type-II multiferroics. In (a), the listed materials show a common feature that the magnetic cation is essentially involved in generating ferroelectricity. In (b), ferroelectric polarization is roughly inversely proportional to *T*_C_ (= *T*_N_) in type-II multiferroics. The gray arrows in both (a) and (b) indicate the target of multiferroic research.

As illustrated in Fig. 11, developed based on our collection of available data, it is clear where the way is for our further efforts (large arrows) while the as-shown dependences are intrinsic in physics. For type-I multiferroics, both *T*_C_ and *T*_N_ can be above room temperature, and electric polarization can reach ∼10 μC/cm^2^ or even larger. However, the ME response is usually weak without much exception. For type-II multiferroics, the remarkable ME coupling cannot prevent our disappointment at the weak ferroelectricity and ferromagnetism. Therefore, to approach the upper-right corners of the diagrams, we need a distinctly different and more promising strategy in single-phase multiferroics.

In comparison with ferroelectricity, magnetism such as net magnetization and *T*_N_ looks much harder to improve in all types of multiferroics. Note that the existence of large macroscopic magnetization is critical for electro-control of magnetism. Regarding this, multiferroics containing more than one type of magnetic moments (due to different elements/valent states/sites etc.) deserve special attention. These various magnetic moments can form either clusters (blocks) or a ferrimagnetic configuration, and thus may allow remarkable macroscopic magnetization without damaging the ferroelectric state, as exemplified in the hexaferrites and the (Mn, Fe)_2_Mo_3_O_8_ family [62,81]. This scenario is not only expected in type-II multiferroics, but also looks compatible with type-I multiferroics, especially for those double-layered perovskites showing hybrid improper multiferroicity. An impressive detail of the hybrid mechanism in orthorhombic RMnO_3_ is the existence of R and Mn, both of which are magnetic, parallel to the scenario proposed to enhance magnetization in multiferroics. Regarding *T*_N_, it is another roadblock. Even for type-I multiferroics, most of them have low *T*_N_ below room temperature. Nevertheless, several routes to enhance *T*_N_ include at least two aspects. First, Fe-based multiferroics usually have a relatively higher *T*_N_ than manganites, probably due to the stronger Fe–Fe exchange [30]. Second, the tolerance factor is pivotal in tuning *T*_N_ in multiferroics, as exemplified in both h-RMnO_3_ and RFeO_3_ [38]. In fact, it was recently found that the tolerance factor has a considerable impact on the properties in double-layered perovskites [94].

Apart from these focused issues, emergent phenomena such as non-reciprocity, giant thermal Hall effect, and topological domain structure are indeed appealing, and have largely broadened the conception of multiferroicity and may bring new functionalities of multiferroics. While these phenomena show fascinating aspects, one may wonder how these effects could be coupled to the various ferroic orders. Furthermore, more emergent and interesting properties hosted in a multiferroic await exploration, and thus certainly more extensive investigations are required to explore the full merit of multiferroics, paving the way for multiferroic research into a new era.
